# Identification and validation of clinical predictors for the risk of neurological involvement in children with hand, foot, and mouth disease in Sarawak

**DOI:** 10.1186/1471-2334-9-3

**Published:** 2009-01-19

**Authors:** Mong How Ooi, See Chang Wong, Anand Mohan, Yuwana Podin, David Perera, Daniella Clear, Sylvia del Sel, Chae Hee Chieng, Phaik Hooi Tio, Mary Jane Cardosa, Tom Solomon

**Affiliations:** 1 Department of Paediatrics, Sibu Hospital, Sibu, Sarawak, Malaysia; 2 Institute of Health and Community Medicine, Universiti Malaysia Sarawak, Kota Samarahan, Sarawak, Malaysia; 3 Division of Neurological Science, Division of Medical Microbiology and Genitourinary Medicine, Liverpool School of Tropical Medicine, University of Liverpool, Liverpool, UK

## Abstract

**Background:**

Human enterovirus 71 (HEV71) can cause Hand, foot, and mouth disease (HFMD) with neurological complications, which may rapidly progress to fulminant cardiorespiratory failure, and death. Early recognition of children at risk is the key to reduce acute mortality and morbidity.

**Methods:**

We examined data collected through a prospective clinical study of HFMD conducted between 2000 and 2006 that included 3 distinct outbreaks of HEV71 to identify risk factors associated with neurological involvement in children with HFMD.

**Results:**

Total duration of fever ≥ 3 days, peak temperature ≥ 38.5°C and history of lethargy were identified as independent risk factors for neurological involvement (evident by CSF pleocytosis) in the analysis of 725 children admitted during the first phase of the study. When they were validated in the second phase of the study, two or more (≥ 2) risk factors were present in 162 (65%) of 250 children with CSF pleocytosis compared with 56 (30%) of 186 children with no CSF pleocytosis (OR 4.27, 95% CI2.79–6.56, p < 0.0001). The usefulness of the three risk factors in identifying children with CSF pleocytosis on hospital admission during the second phase of the study was also tested. Peak temperature ≥ 38.5°C and history of lethargy had the sensitivity, specificity, positive predictive value (PPV) and negative predictive value (NPV) of 28%(48/174), 89%(125/140), 76%(48/63) and 50%(125/251), respectively in predicting CSF pleocytosis in children that were seen within the first 2 days of febrile illness. For those presented on the 3^rd ^or later day of febrile illness, the sensitivity, specificity, PPV and NPV of ≥ 2 risk factors predictive of CSF pleocytosis were 75%(57/76), 59%(27/46), 75%(57/76) and 59%(27/46), respectively.

**Conclusion:**

Three readily elicited clinical risk factors were identified to help detect children at risk of neurological involvement. These risk factors may serve as a guide to clinicians to decide the need for hospitalization and further investigation, including cerebrospinal fluid examination, and close monitoring for disease progression in children with HFMD.

## Background

Hand, foot, and mouth disease (HFMD) is a common childhood exanthema caused by species A human enteroviruses (HEVA), particularly Coxsackievirus A16 (CVA16)[1]. In most instances, this is a mild self-limiting illness. The affected children are often given out-patient care with symptomatic treatment. However over the last decade HFMD has emerged as a growing public health problem in Asia following frequent outbreaks of death-associated HFMD caused by a more virulence member of HEVA, human enterovirus 71 (HEV71), in a number of countries in the region [2-5]. This was first recognized with large outbreaks of HFMD associated with neurological disease and alarming fatalities in Sarawak, Malaysia in 1997 and in Taiwan in 1998 [2,3]. Fatal cases typically presented with a brief duration of febrile illness, subtle neurological signs and died dramatically of acute refractory cardiac dysfunction and fulminant pulmonary oedema within hours of developing signs of tachycardia, poor peripheral perfusion and tachypnea. Indeed, most of them died shortly after hospital admission, and some even before or on arrival at hospital [2,6-8]. Although severe neurological complications and death only occur in a small minority of children with HFMD, the fulminant disease course of the fatal cases has caused great public alarm in Asia. Experience from recent outbreaks of HEV71 associated HFMD (HEV71-HFMD) in Asia showed that primary care doctors are often overwhelmed with large number of children with HFMD seeking medical attention for the fear of neurological complications and death. Because of the risk of sudden death, coupled with tremendous parental pressure to admit children with HFMD into hospital for observation, children with HFMD are often routinely admitted into hospital for observation in Sarawak, which has imposed a huge burden on the healthcare system. Cerebrospinal fluid (CSF) pleocytosis has so far been the universal finding in fatal cases even though many have no obvious neurological signs prior to sudden onset of cardiorespiratory failure and death [2,6,8]. In the absence of clear neurological sign, CSF pleocytosis (indicative of neurological involvement) has thus been considered an objective marker of complicated disease, allowing clinicians to focus their attention and provide timely intervention in these patients before they develop fatal cardiorespiratory failure. We therefore examined data collected through a prospective study of HFMD to identify and validate risk factors associated with neurological involvement in children with HFMD that may be used by clinicians managing children with HFMD.

## Methods

### Setting and study period

A prospective clinical study was conducted from January 2000 through December 2006, which included 3 distinct outbreaks that occurred in 2000/1, 2003 and 2006, at the paediatric wards and intensive care unit at Sibu Hospital (Sarawak, Malaysia). The study was approved by the Director of Health for Sarawak and the Ethics Committee of the Liverpool School of Tropical Medicine (UK). Informed consent was obtained verbally from each child's accompanying parent or guardian.

### Case definitions

**Figure 1:**
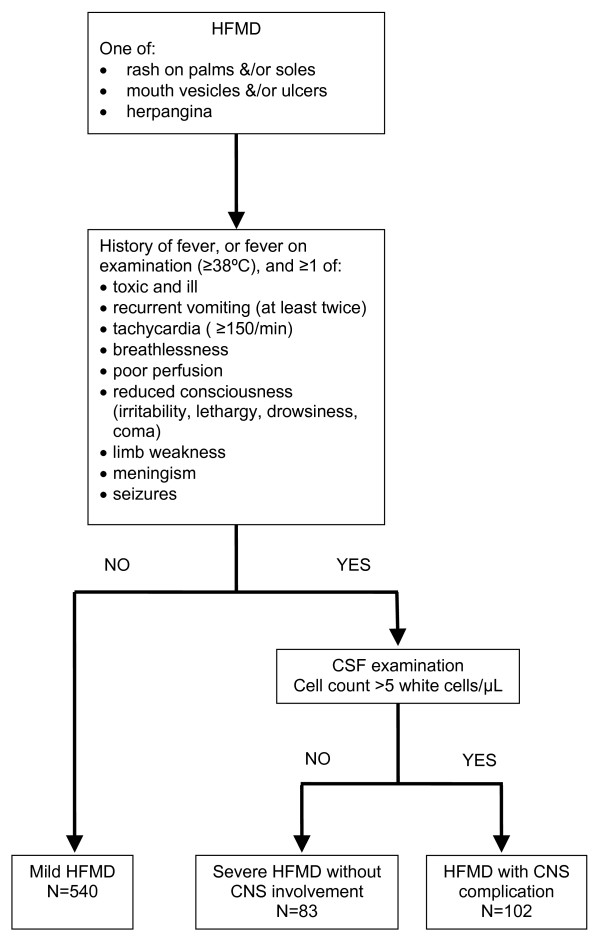
**Case definitions**. The flow chart shows the algorithm of the investigation and the classification of the disease severity of children with HFMD used in the study. HFMD: Hand, foot, and mouth disease, CSF: Cerebrospinal fluid, CNS: Central nervous system.

Figure 1 shows the algorithm of the investigation and the classification of the disease severity of children with HFMD in the study [9]. A child was defined as having HFMD if they had new onset of at least one (≥ 1) of the following: maculopapular or vesicular rash on the palms and/or soles; vesicles or ulcers in the mouth or herpangina (defined as multiple oral ulcers predominantly affecting the posterior parts of the oral cavity). Children with HFMD were considered to have more serious illness if they have the following features: a history of fever, or fever on examination (≥ 38°C), and ≥ 1 of the following features indicative of more serious illness: toxic and ill in appearance, recurrent vomiting (at least twice), tachycardia (heart rate ≥ 150/min) breathlessness, poor perfusion (cold clammy skin), reduced consciousness (irritability, lethargy, drowsiness, coma), limb weakness, meningism (neck stiffness or positive Kernig's sign), seizures. They were subjected to CSF examination after written consent to exclude central nervous system (CNS) involvement. Children with > 5 cells/μL (i.e. CSF pleocytosis) and negative microscopy and culture for bacteria were classified as "HFMD with CNS complications" (HFMD-CNS), while those with normal CSF examination were considered to have "severe HFMD without CNS involvement" (HFMD-Non-CNS). Children with HFMD-CNS were diagnosed to have aseptic meningitis (ASM) if they were fully conscious, had headache, meningism, and no focal neurological signs. Encephalitis was defined by the presence of impaired consciousness including lethargy, drowsiness or coma, seizures or myoclonus. Acute flaccid paralysis (AFP) was characterized by the acute onset of areflexic limb weakness. Cardiorespiratory failure was defined by the presence of tachycardia, respiratory distress, pulmonary oedema, poor peripheral perfusion requiring inotropes, pulmonary congestion on chest radiography and reduced cardiac contractility on echocardiography. Children without features of more serious illness were classified as "mild HFMD", and were observed in hospital until they became afebrile for at least 12–24 hours. A child was considered to be positive for HEV71 if HEV71 was isolated by tissue culture or HEV71 RNA was detected by HEV71 specific RT-PCR from ≥ 1 clinical sample.

### Clinical methods

All children with HFMD admitted into the hospital were assessed by pediatricians of the study team. A detailed history and clinical examination was performed with special attention to mucocutaneous lesions, cardiovascular and neurological signs. All details were recorded on standardized forms. Swabs were taken from the throat and rectum of every patient, as well as ≥ 1 swab from vesicles on the skin and oral ulcers (if present). The clinical samples were stored immediately in a -70°C freezer until further testing. Blood was taken for flavivirus serology, and in patients with suspected CNS involvement for full blood count, urea, electrolytes, and glucose. Electrocardiogram and echocardiogram was also performed on children with suspected CNS involvement. CSF was examined for cell count and differential, protein, glucose, Gram stain, bacterial culture and processed for viral studies. If there was a strong clinical suspicion of viral CNS infection, but the initial CSF examination was acellular, a second lumbar puncture was performed. Lumbar punctures were delayed in those with unstable vital signs. Patients were examined daily or more frequently as indicated, by a member of the study team. Children with HFMD-CNS complications (particularly those with encephalitis and acute flaccid paralysis) were treated with intravenous immunoglobulin (IVIG) at the discretion of the treating physician [10].

### Virological methods

Virus isolation was attempted on all swab specimens, CSF specimens, and any serum samples remaining after other investigations had been completed through the inoculation of human rhabdomyosarcoma and human embryonic kidney cells. Isolated enteroviruses were typed by nucleotide sequencing of VP1 and VP4 genes and genogrouped by phylogenetic analysis [11,12]. During the 2006 outbreak, in addition to virus isolation, all swab specimens were also tested for presence of HEV71 RNA using a HEV71 specific RT-PCR [13]. Paired serum samples (obtained on the day of admission and on day 7, or on the day of discharge or after death) and CSF specimens were also tested for IgM against dengue and Japanese encephalitis virus (JEV) in parallel, using an IgM-capture ELISA that distinguishes responses to these two viruses [14].

### Statistical analysis

Data from HFMD patients recruited in the first phase of the study (mostly during 2 outbreaks that occurred between January 2000 and July 2003) were used to identify risk factors for neurological involvement (evident by CSF pleocytosis). The primary analysis was for variables associated with neurological involvement by comparing children with HFMD-CNS (i.e. with CSF pleocytosis) to those with HFMD-Non-CNS (i.e. no CSF pleocytosis). Variables that were considered potentially useful to primary care doctors in identifying children with neurological involvement were included in a multiple logistic regression analysis to look for independent risk factors for neurological involvement (i.e. CSF pleocytosis). Variables were selected backward and remained in the model only if they were statistically associated with neurological involvement (p < 0.05). (SPSS software, Version 13.0; SPSS). The association between the independent risk factors identified and neurological involvement were validated in the second phase of the study, where most patients were admitted during the 2006 outbreak. The utility of the identified risk factors as clinical predictors for neurological involvement at the point of first contact for care was also examined. Normally distributed data were compared using Student's *t *test; data that were not normally distributed were compared by the Mann-Whitney *U *test (Statview 4.02; Abacus Concepts). Differences between proportions were tested using the Chi-square test with Yates's correction or Fisher's exact test as appropriate (Epi Info, version 6; Centers for Disease Control and Prevention). A p value < 0.05 was considered statistically significant.

## Results

A total of 725 children (457, 63% males) were recruited between 1^st ^January 2000 and 31^st ^July 2003. Most children were recruited during 2 large outbreaks of HEV71-HFMD that occurred during 2000/2001 and 2003. Five hundred and forty (74%) children had mild HFMD. One hundred and eighty five (26%) children had suspected CNS involvement and required CSF examination; 102 (55%) of them had CSF pleocytosis (HFMD-CNS) and the remaining 83 (45%) had normal CSF findings (HFMD-Non-CNS). Of the 102 children with HFMD-CNS, 63 (62%) had ASM, 33 (32%) had encephalitis, 3 (3%) had AFP, and 3 (3%) had encephalitis associated with cardiorespiratory failure (all of the 3 died). Of the 273 HEV71 culture positive children, 187 (69%) had mild HFMD, 34 (13%) had HFMD-Non-CNS, and 52 (19%) had HFMD-CNS (30 had ASM, 19 had encephalitis, 1 had AFP and 2 had encephalitis with cardiorespiratory failure). Detailed results of the epidemiology, diagnostic virology and molecular epidemiology of this phase of the study have been reported previously [9,15].

### Clinical features

#### Comparison of patients with HFMD with CNS complications with those had more serious HFMD without CNS involvement (January 2000 to July 2003)

The clinical features of the children with HFMD-CNS (i.e. with CSF pleocytosis) are compared to those with HFMD-Non-CNS (i.e. no CSF pleocytosis) (see Additional file 1). Children with HFMD-CNS were more likely to be male and of Chinese ethnic group. Children of Iban ethnic group, however, were less likely to have HFMD with CNS complications. Children with HFMD-CNS complications were more likely to have higher mean peak temperature, peak temperature ≥ 38.5°C, longer mean total duration of fever and total duration of fever ≥ 3 days. Findings of lethargy (from the parent's history or physical examination), faster mean heart rate, mean heart rate ≥ 150/min and limb weakness on examination were more frequently present in children with HFMD-CNS. There was no difference in the proportion of children with positive HEV71 isolation between children with HFMD-CNS and those with HFMD-Non-CNS.

To look for independent risk factors that could be used to predict neurological involvement evident by CSF pleocytosis, total duration of fever ≥ 3 days, peak temperature ≥ 38.5°C, being lethargic (from the parent's history or physical findings), history of breathlessness, history of vomiting, history of or witnessed myoclonus, neck stiffness were included in a multiple logistic regression analysis. Total duration of fever ≥ 3 days, peak temperature ≥ 38.5°C and history of lethargy were found to be independent risk factors of neurological involvement after multivariate analysis (Table 1). Table 2 shows the number and type of the risk factors that were present in the 725 children with HFMD seen during the first phase of the study according to the disease severity. Two or more (≥ 2) risk factors were present in 83% (85/102) of patients that had HFMD-CNS when compared to 43% (36/83) of patients with HFMD-Non-CNS (OR 6.53, 95% CI 3.15–13.66, p < 0.0001). Further analysis on the HEV71-positive subset showed that ≥ 2 risk factors were present in 82% (43/52) of children with HFMD-CNS when compared to 32% (11/34) patients with HFMD-Non-CNS (OR 9.99, 95%CI 3.26–31.82). A separate analysis on children with mild HFMD showed that ≥ 2 risk factors were present in 6% of cases with mild HFMD (32/540)(Table 2), and HEV71-postive mild HFMD (11/187), respectively.

**Table 1:** Risk factors that were significantly associated with CSF pleocytosis in children with HFMD in the first phase of the study (2000 to 2003).

Risk factors	p value	Odds ratio	95% CI
Total duration of fever ≥ 3 days	< 0.0001	6.52	2.83 – 14.99
Peak temperature ≥ 38.5°C	0.0192	2.27	1.14 – 4.51
History of lethargy	0.001	3.18	1.60 – 6.35

Note: The Hosmer-Lemeshow statistics indicated a non-significance of lack of fit (χ^2 ^= 2.163, p = 0.904).

CSF: Cerebrospinal fluid

HFMD: Hand, foot, and mouth disease

**Table 2:** The number and type of the risk factors that were present in the children with HFMD seen in the 2000/3 and 2006 outbreaks

**Risk factors that were present**	**First phase of study (2000/3)**			**Second phase of study (2006)**		
		
	**HFMD-CNS (N = 102)**	**HFMD-Non-CNS (N = 83)**	**Mild HFMD** **(N = 540)**	**HFMD-CNS** **(N = 250)**	**HFMD-Non-CNS (N = 186)**	**Mild HFMD** **(N = 294)**
	
**No. of patients with none of the 3 risk factor**	**2**	**9**	**352**	**11**	**52**	**208**
	
						
	
Peak temperature ≥ 38.5°C only	1	8	7	29	32	34
History of lethargy only	5	15	27	16	11	17
Total duration of fever ≥ 3 only	9	15	122	32	35	30
	
**No. of patients with 1 risk factor**	**15**	**38**	**156**	**77**	**78**	**81**
						
	
Peak temperature ≥ 38.5°C plus history of lethargy	2	3	1	11	7	2
Peak temperature ≥ 38.5°C plus total duration of fever ≥ 3 days	25	15	8	76	31	3
Total duration of fever ≥ 3 days plus history of lethargy	26	12	19	21	5	0
	
**No. of patients with 2 risk factors**	**53**	**30**	**28**	**108**	**43**	**5**
						
	
**No. of patients with 3 risk factors (Peak temperature ≥ 38.5°C plus total duration of fever ≥ 3 days plus history of lethargy)**	**32**	**6**	**4**	**54**	**13**	**0**

Note:

HFMD: Hand, foot, and mouth disease

HFMD-CNS: Hand, foot, and mouth disease with central nervous system complication

HFMD-Non-CNS: Severe HFMD without central nervous system involvement

#### Validation of the association between the risk factors and neurological involvement in children with HFMD in 2006 outbreak

The association between the identified risk factors (total duration of fever ≥ 3 days, peak temperature ≥ 38.5°C and history of lethargy) and neurological involvement were validated in the 2006 outbreak. A total of 730 children with HFMD were admitted between January and December 2006. Two hundred and ninety four (40%) children had mild HFMD. Four hundred and thirty six (60%) children had features of more serious illness and warranted CSF examination; 250 (34%) of them had HFMD-CNS and the remaining 186 (26%) had HFMD-Non-CNS. Of the 250 children with HFMD-CNS, 65 (26%) had ASM, 172 (69%) had encephalitis, 2 (0.8%) had encephalitis associated with AFP, and 11 (4.4%) had encephalitis associated with cardiorespiratory failure (6 of them died). HEV71 was isolated from 157 (27%) of 586 children who had virus isolation done. A further 44 (7%) children had other HEVA (n = 29) and species B HEV (n = 15). No patient had CVA16 isolated. HEV71RNA was detected in 239 (50%) of 477 children that were tested with HEV71 specific RT-PCR. In short, 291 (45%) of 653 children were positive for HEV71. Of the 291 HEV71-positive children, 104 (36%) had mild HFMD, 73 (25%) had HFMD-Non-CNS, 114 (39%) had HFMD-CNS (22 had ASM, 83 had encephalitis, 2 had encephalitis associated with AFP, 7 had encephalitis associated with cardiorespiratory failure). HEV71 was detected in 4 (67%) of the 6 fatal case children that had encephalitis associated with cardiorespiratory failure. The Additional file 2 shows the clinical features of the 730 children that were admitted during the 2006 outbreak according to the disease severity. Total duration of fever ≥ 3 days, peak temperature ≥ 38.5°C and history of lethargy were similarly more frequently present in children with HFMD-CNS than those with HFMD-Non-CNS. Two or more risk factors were present in 65% (162/250) of children that had HFMD-CNS when compared with 30% (56/186) of children with HFMD-Non-CNS (OR 4.27, 95%CI 2.79–6.56, p < 0.0001) (Table 2). Among children with HEV71-positive HFMD, ≥ 2 risk factors were present in 61% (69/114) of children with HFMD-CNS when compared with 26% (19/73) of children with HFMD-Non-CNS (OR 4.36, 95%CI 2.19–8.75, p < 0.0001). A separate analysis on children with mild HFMD showed that history of lethargy, total duration of fever ≥ 3 days and peak temperature ≥ 38.5°C was present in 6.4% (19/294), 11% (33/294) and 14% (42/294) of the children with mild HFMD, respectively (Additional file 2). Two or more 2 risk factors were found in only 5 (2%) of 294 with mild HFMD (Table 2) and in 1 (1%) of 104 of children with HEV71-positive mild HFMD.

#### The usefulness of the risk factors in predicting neurological involvement in children with HFMD in the 2006 outbreak

**Figure 2:**
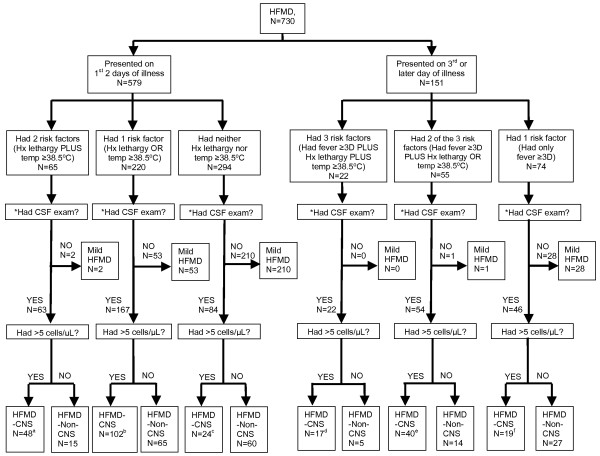
**Classification of 730 Children with HFMD**. The flow chart shows the distribution and classification of disease severity of 730 children with HFMD in the 2006 outbreak according to the duration of fever and the risk factors that were present when they first presented to the hospital. CSF examination is indicated if the children have features indicative of more serious illness of HFMD (see case definition in main text). Hx lethargy: History of lethargy, Temp ≥ 38.5°C: body temperature ≥ 38.5°C, CSF exam: cerebrospinal fluid examination, HFMD: Hand, foot, and mouth disease, HFMD-CNS: Hand, foot, and mouth disease with central nervous system complication, HFMD-Non-CNS: Severe HFMD without central nervous system involvement, BENC: brainstem encephalitis, ASM: aseptic meningitis. a. Of the 48 children with HFMD-CNS, 40 had BENC, 6 had ASM and 2 had BENC associated with cardiorespiratory failure (1 of whom died). b. Of the 102 children with HFMD-CNS, 74 had BENC, 26 had ASM, 1 had encephalitis and 1 had fatal BENC associated with cardiorespiratory failure. c. Of the 24 children with HFMD-CNS, 13 had BENC, 8 had ASM, 1 each had BENC associated with cardiorespiratory failure, encephalitis, and encephalitis associated with acute flaccid paralysis. d. Of the 17 children with HFMD-CNS, 11 had BENC, 5 had BENC associated with cardiorespiratory failure (4 of whom died) and 1 had ASM. e. Of the 40 children with HFMD-CNS, 22 had BENC, 17 had ASM and 1 had fatal BENC associated with cardiorespiratory failure. f. Of the 19 children with HFMD-CNS, 10 had BENC, 7 had ASM and 1 each had encephalitis with acute flaccid paralysis, and BENC associated with cardiorespiratory failure.

We were particularly interested to assess the utility of the three clinical risk factors in predicting neurological involvement in children with HFMD at the point of first contact for care. While a febrile illness ≥ 3 day was an important risk factor for severity, primary care physicians often see many children on the first 2 days of HFMD illness. To determine if peak temperature ≥ 38.5°C and history of lethargy are useful in identifying children who sought treatment within the first 2 days of the febrile illness we performed a separate analysis for children who presented within the first 2 days of the illness during the 2006 outbreak. Figure 2 shows the distribution and classification of disease severity of 730 children with HFMD in the 2006 outbreak according to the duration of febrile illness and the risk factors that were present when they first presented to hospital. Five hundred and seventy nine (79%) of 730 children were admitted within the first 2 days of febrile illness. Sixty five (11%) of the 579 children had history of lethargy plus peak temperature ≥ 38.5C. All but two (97%) of the 65 children had features of more serious illness and warranted CSF examination. About three quarter of them had CSF pleocytosis and was classified as HFMD-CNS. Only 2 (3%) of the 65 children were labeled as mild HFMD. Two hundred and twenty (38%) children had only either history of lethargy or peak temperature ≥ 38.5C. Of the 167 (76%) children who warranted a CSF examination, 102 (61%) of them had CSF pleocytosis, and were classified as HFMD-CNS. The remaining 53 (24%) children without feature of more serious illness were considered as mild HFMD. Two hundred and ninety four (51%) children had neither of the two risk factors. Eighty four (29%) of the 294 children, however, had other features of more serious illness, and hence underwent CSF examination. CSF pleocytosis was found in 24 (29%) of the 84 children, and were classified as HFMD-CNS. Two hundred and ten (71%) of the 294 children without features of more serious illness were labeled as mild HFMD. In summary CSF pleocytosis was found in 48(74%) of 65 children with 2 risk factors (temperature ≥ 38.5°C and history of lethargy) on hospital admission compared with that in 126 (25%) of 514 children with ≥ 1 risk factors (OR 8.69; 95%CI 4.66–16.37, p < 0.0001).

One hundred and fifty one (21%) of the 730 children were seen on the 3^rd ^or later days of their febrile illness. Twenty two (15%) of the 151 children had all the 3 risk factors associated with neurological involvement. All the 22 children warranted CSF examination. Seventeen (77%), including 4 fatal cases, of the 22 children had CSF pleocytosis and were classified as HFMD-CNS. Of the 55 (36%) children that had 2 risk factors, all except one child required CSF examination to exclude CNS involvement. Forty (74%) of the 54 children had CSF pleocytosis and were classified as HFMD-CNS. Being febrile for ≥ 3 days was the sole risk factor in 74 (49%) of the 151 children. Forty six (62%) children had features of more serious illness, and underwent CSF examination – 19 (41%) had CSF pleocytosis and were classified as HFMD-CNS. The remaining 28 (38%) children were labeled as mild HFMD. In short CSF pleocytosis was found in 57(74%) of 77 children that had ≥ 2 risk factors on hospital admission compared with in 19 (26%) of 74 children with isolated risk factor of being febrile ≥ 3 days (OR 8.25; 95%CI 3.75–18.38, p < 0.0001). Further analysis on the HEV71-positive subset showed that 24% (21/86) of children with HFMD-CNS presented within the first 2 days of febrile illness had ≥ 2 risk factors compared with 10% (6/60) of children with HFMD-Non-CNS (OR 2.91; 95% CI 1.03–9.38, p = 0.0464). For the HEV71-positive children presented on the 3^rd ^or later days of febrile illness, 71% (20/28) of children with HFMD-CNS had ≥ 2 risk factors compared with 31% (4/13) of children with HFMD-Non-CNS (OR 5.63; 95% CI 1.11–31.35, p = 0.0341). The sensitivity, specificity, positive predictive value and negative predictive value of the risk factors in predicting CSF pleocytosis in children with HFMD at presentation in the 2006 outbreak is shown in Table 3.

**Table 3:** The sensitivity, specificity, positive predictive value and negative predictive value of the risk factors.

Risk factors that were present	Presented within the first 2 days of febrile illness				Presented on the 3rd or later day of febrile illness			
		
	Sensitivity	Specificity	PPV	NPV	Sensitivity	Specificity	PPV	NPV
	
Both peak temperature ≥ 38.5°C and history of lethargy	28% (48/174) [23–33%]	89% (125/140) [86–92%]	76% (48/63) [71–81%]	50% (125/251) [44–56%]	22% (17/76) [15–29%]	89% (41/46) [83–95%]	77% (17/22) [70–84%]	41% (41/100) [32–50%]
	
Peak temperature ≥ 38.5°C &/or history of lethargy	86% (150/174) [82–90%]	43% (60/140) [38–48%]	65% (150/230) [60–70%]	71% (60/84) [66–76%]	75% (57/76) [67–83%]	59% (27/46) [50–68%]	75% (57/76) [67–83%]	59% (27/46) [50–68%]

(%, proportion, [95% confidence interval]) in predicting CSF pleocytosis in the 436 children with suspected CNS involvement seen in the 2006 outbreak

Note:

CSF: Cerebrospinal fluid

CNS: Central nervous system

Sensitivity = The proportion of children with CSF pleocytosis that are correctly identified by the presence of the risk factors

Specificity = The proportion of children without CSF pleocytosis that are correctly identified by the absence of the risk factors

Positive predictive value (PPV) = The proportion of individuals with the risk factors that have CSF pleocytosis

Negative predictive value (NPV) = The proportion of individuals without the risk factors that do not have CSF pleocytosis

Between 2000 and 2006, a total of 352 children with CNS involvement were admitted into the study. One hundred and twenty eight (36%) children had ASM (a mild and benign CNS involvement) and 224 (64%) had severe and potentially fatal CNS complications (205 had encephalitis, 14 had encephalitis associated with cardiorespiratory failure, 2 had encephalitis associated with AFP, 3 had AFP). Among the 224 children that had severe CNS complications, 204 (95%) of 215 children that survived had timely hospital admission and IVIG treatment compared to one (11%) of 9 children that died (OR 148.36, 95%CI 16.34–6609.04, p < 0.0001). Table 4 shows the clinical details and the risk factors that were present in the 9 fatal case children on hospital admission. Two or more risk factors associated with neurological involvement were present in all the 9 fatal children, and were noted for ≥ 24–48 hours before hospital admission.

**Table 4:** The clinical details and risk factors for neurological involvement of the nine fatal case children with HFMD seen in the study.

Patient	Year of the outbreak	Age (months)	Day of illness at presentation	Risk factors that were present at presentation	Disease severity	HEV71 detected?	IVIG treatment	Note
1	2000	11	Day 3	fever ≥ 3D, history of lethargy, temperature ≥ 38.5°C	HFMD-CNS	Yes	No	a.
2	2003	34	Day 5	fever ≥ 3D, history of lethargy	HFMD-CNS	Yes	No	b.
3	2003	32	Day 3	fever ≥ 3D, history of lethargy	HFMD-CNS	No	No	a.
4	2006	9	Day 1	history of lethargy, temperature ≥ 38.5°C	HFMD-CNS	Yes	Yes	c.
5	2006	8	Day 3	fever ≥ 3D, history of lethargy, temperature ≥ 38.5°C	HFMD-CNS	Yes	No	a.
6	2006	14	Day 3	fever ≥ 3D, history of lethargy, temperature ≥ 38.5°C	HFMD-CNS	Yes	No	a.
7	2006	34	Day 4	fever ≥ 3D, history of lethargy, temperature ≥ 38.5°C	HFMD-CNS	Yes	No	a.
8	2006	25	Day 4	fever ≥ 3D, history of lethargy, temperature ≥ 38.5°C	HFMD-CNS	No	No	a.
9	2006	47	Day 4	fever ≥ 3D, history of lethargy	HFMD-CNS	No	No	a.

Note:

a. Presented in the moribund state with fulminant cardiorespiratory failure and pulmonary oedema. The patient died within 24 hours of the hospitalization. The risk factors were present for ≥ 48 hours before hospital admission.

b. Developed acute cardiorespiratory collapse and died 12 hours after hospitalization. Had peak temperature ≥ 38.5°C in the hospital. The patient was lethargic for ≥ 48 hours before hospital admission.

c. Deteriorated progressively because of cardiorespiratory failure despite intensive care support. Died on day 4 of the hospitalization. The patient was lethargic for 24 hours before hospital admission

HFMD-CNS: Hand, foot and mouth disease with central nervous system complication

IVIG: Intravenous immunoglobulin

## Discussion

Early recognition of children at risk of neurological involvement and death (particularly those with encephalitis and encephalomyelitis) is critical as the disease progression from the onset of neurological involvement to fulminant cardiorespiratory failure may be remarkably rapid [8]. However the clinical manifestations of neurological involvement may be very subtle, particularly in young children with early CNS disease [8,16]. While the signs of cardiorespiratory distress such as breathlessness, tachypnea, tachycardia, poor perfusion are easy to recognize, they invariably appear very late shortly before most fatal case collapsed. Our results and other published studies showed that timely diagnosis and intervention, including the use of IVIG infusion, may reduce acute mortality [10,17-19]. Hence the primary care doctors are confronted with a clinical challenge of identifying a small fraction of children who are at risk of neurological complication from an overwhelmingly large number of children who would have uncomplicated course of HFMD. For this reason it is important to find clinical predictors for neurological involvement that can guide primary care doctors perform a proper patient triage, which should be aimed to admit high risk children into hospital early for close observation and further management, while those at low risk of neurological complication may be given out-patient care after parental education and advice. Few studies have systemically examined how to identify children at risk early before they develop cardiorespiratory failure, particularly at the primary care setting where the majority of children with HFMD would first seek treatment during a community outbreak of HFMD.

In this study we identified and validated that history of lethargy, mean peak temperature ≥ 38.5°C and total duration of fever ≥ 3 days were important risk factors for neurological involvement. Our study also shows that neurological involvement occurs at early course of complicated HFMD, and may be detectable within the first 2 days of the febrile illness because CSF pleocytosis was present in 174 (30%) of 579 children seen within the first 2 days of febrile illness, where they also formed 70% (174/250) of children with HFMD-CNS in the 2006 outbreak (Figure 2). Since CSF pleocytosis may be detectable within the first 2 days of the febrile illness and fulminant cardiorespiratory failure seen in the fatal case children typically occurred on the 3^rd ^or later day of febrile illness, it is imperative to attempt to identify children at risk of neurological involvement before the 3^rd ^day of febrile illness so that they can be admitted into hospital early for close monitoring and investigation, and intervention may be instituted when necessary.

Examination of body temperature and careful enquiry into history of lethargy, duration of fever and home record of body temperature should form an integral part of HFMD patient triage at the primary care level. The three risk factors are readily elicited, and can also be used after minimal training by paramedics, who are the key primary care providers in many developing countries including in Sarawak (Malaysia) in Asia. The parents of children with HFMD can also play an important role in early diagnosis of neurological complication in children with HFMD. They should be educated about the 3 risk factors, and be encouraged to monitor the children's body temperature regularly and observe the children's physical activity closely. Body temperature ≥ 38.5°C and history of lethargy may be particularly useful clinical clues for neurological involvement during the first 2 days of febrile illness since at this time the presentation of complicated HFMD is typically undifferentiated and subtle, even to the experienced clinicians [8]. Indeed both history of lethargy and temperature ≥ 38.5°C were observed for 24–48 hours in all the 9 fatal case children before they succumbed to unexpected fulminant cardiorespiratory failure (Table 4). Primary care doctors should have high index of suspicions of neurological complication when they are presented with children with HFMD who have been febrile ≥ 3 days. The children should be admitted into hospital for close observation and investigated for CNS involvement, if necessary. Our study showed that 92 (31%) of 293 children with total duration of fever ≥ 3 days in the 2000/3 outbreak, and 183 (61%) of 300 children in the 2006 outbreak had neurological involvement (Table 2). CSF pleocytosis was present in 25% (19/74) of children with a single risk factor of being febrile ≥ 3 days on hospital admission (Figure 2). The risk of CNS complication is increased significantly when there are added risk factors of having history of lethargy and temperature ≥ 38.5°C. In contrast children who have a brief duration of low grade fever (≥ 38.5°C) and no history of lethargy are of low risk of neurological disease, and may be provided with out-patient care and parental reassurance.

Our results are in keeping with findings reported by Chang and co-authors where fever ≥ 39°C, fever duration ≥ 3 days and lethargy were more frequently observed in children with CNS involvement and in children with HEV71-HFMD than in those with CVA16-HFMD [8,20]. Although several other clinical features and laboratory abnormalities have been associated with fatal HEV71-HFMD, they have yet been validated, and been shown useful in detecting neurological disease or disease progression [8,21,22]. For example, Chong and co-authors reported that absence of mouth ulcers predicted a more complicated or fatal HFMD, and have recommended that children without mouth ulcers should be monitored closely [22]. However, in our study we did not find that children without mouth ulcers were more likely than children with mouth ulcers to have features of more severe HFMD or develop neurological complication. Not all the risk factors identified in these published studies can readily be translated into clinical practice, particularly at primary care settings. Hyperglycemia and leucocytosis have been shown as risk factors for fatal HEV71 disease [8]. However, in our experience hyperglycemia and leucocytosis are late laboratory changes in children with fulminant cardiorespiratory failure (unpublished observations), and thus are not helpful clinically in identifying children at high risk of complication and death. Elevated cardiac Troponin I, a sensitive cardiac-specific biomarker for myocardial injury, has been noted in children who subsequently developed left ventricular failure, and may be useful in identifying patients at risk of left ventricular failure and pulmonary oedema [21]. Although cardiac Troponin I has been used widely in developed countries for early diagnosis of acute coronary syndrome, it is expensive and not widely available in many developing countries, including in Sarawak. Screening for heart rate variability abnormalities, an index of autonomic nervous system, through non-invasive continuous ECG monitoring may provide early warning of impending cardiorespiratory failure about 7 hours before its onset [16]. The labour-intensive approach is most suited in a critical care setting for children already diagnosed of CNS involvement because it requires the use of expensive and sophisticated device, and close monitoring for the heart rate abnormalities.

A limitation of our study is that the clinical predictors developed for use at primary care setting were identified and tested using data collected through a hospital-based study. Clinical characteristics of children treated at primary care settings may differ from hospitalized children. However, as a large number of children with mild HFMD were admitted into our study, we had the opportunity to systemically examine the clinical feature of HFMD of varying severity, including children with mild disease that would normally be treated at primary care clinics, where we have shown history of lethargy, peak temperature 38.5C and total duration of fever 3 days were reported infrequently in children with mild HFMD.

## Conclusion

Currently there is no vaccine against HEV71 infection. Early recognition of children at risk of fulminant pulmonary oedema and cardiac dysfunction is the key to reduce acute mortality and morbidity. We have identified three clinical risk factors that may help detect children at risk of neurological involvement and death at primary care settings, which can guide primary care doctors decide if hospital admission is warranted when they see with children with HFMD. These risk factors are readily elicited through history taking and measurement of body temperature. They may also provide useful guide to help clinicians to decide the need for CSF examination, as well as to monitor disease progression in children with HFMD.

## Competing interests

The authors declare that they have no competing interests.

## Authors' contributions

MHO, SCW, MJC and TS conceived of the study; they were assisted by AM, CHC, DC and SS in the planning, design, and execution of the clinical aspects and by PHT, YP and DP in the analysis and interpretation of the clinical samples; all authors contributed to writing the manuscript.

## Pre-publication history

The pre-publication history for this paper can be accessed here:

http://www.biomedcentral.com/1471-2334/9/3/prepub

## Supplementary Material

Additional file 1 **Clinical features of the 725 children with Hand, foot and mouth Disease that were admitted between January 2000 and July 2003 according to the clinical severity.** The clinical features of the children with HFMD-CNS (i.e. with CSF pleocytosis) are compared to those with HFMD-Non-CNS (i.e. no CSF pleocytosis). The clinical features of children with mild HFMD is also included here. Click here for file

Additional file 2 **Clinical features of the 730 children with Hand, foot and mouth Disease that were admitted during the 2006 outbreak according to clinical severity.** The clinical features of the children with HFMD-CNS (i.e. with CSF pleocytosis) are compared to those with HFMD-Non-CNS (i.e. no CSF pleocytosis). The clinical features of children with mild HFMD is also included here. Click here for file
