# Supplementary material for: Identification and validation of clinical predictors for the risk of neurological involvement in children with hand, foot, and mouth disease in Sarawak
Source: BMC Infect Dis. 2009 Jan 19;9:3. doi: 10.1186/1471-2334-9-3 (PMC2637878; doi:10.1186/1471-2334-9-3)
Supplement: Additional file 1 — Clinical features of the 725 children with Hand, foot and mouth Disease that were admitted between January 2000 and July 2003 according to the clinical severity. The clinical features of the children with HFMD-CNS (i.e. with CSF pleocytosis) are compared to those with HFMD-Non-CNS (i.e. no CSF pleocytosis). The clinical features of children with mild HFMD is also included here. [file 1471-2334-9-3-S1.doc]

| Additional File 1. Clinical features of 725 children with hand, foot, and mouth disease that were admitted between January 2000 and July 2003 according to the clinical severity | | | | | |
| --- | --- | --- | --- | --- | --- |
| Severity | HFMD-CNS (with CSF pleocytosis) | HFMD-Non-CNS (no CSF pleocytosis) | p value^*^ |  | Mild HFMD |
| Number of children | 102 | 83 | -- |  | 540 |
| **History** |  |  |  |  |  |
| Median age in months (range) | 25 [5-120] | 25 [5-104] | 0.7582 |  | 29 [2-153] |
| Male, no (%) | 73 [71.6%] | 47 [56.6%] | 0.0497 |  | 337 [62.4%] |
| Chinese | 44 [43.1%] | 22 [26.5%] | 0.0282 |  | 209 [38.7%] |
| Iban | 28 [27.5%] | 46 [55.4%] | 0.0002 |  | 188 [34.8%] |
| Malay/Melanau | 30 [29.4%] | 15 [18.1%] | 0.1062 |  | 125 [23.1%] |
| Fever at home | 100 [98%] | 77 [92.8%] | 0.1425 |  | 402 [74.4%] |
| Mean duration of fever at home (days) | 2.6 [0-8] | 2.0 [0-7] | 0.0078 |  | 1.6 [0-7] |
| Mean total duration of fever (days) | 4.5 [1-12] | 2.9 [0-8] | <0.0001 |  | 1.8 [0-10] |
| Total duration of fever ≥ 3 days | 92 [90.2%] | 48 [57.8%] | <0.0001 |  | 153 [28.3%] |
| Past history of HFMD | 3 [2.9%] | 3 [3.6%] | >0.9999 |  | 12 [2.2%] |
| Had history of contact with children with HFMD | 34 [33.3%] | 21 [25.3%] | 0.3044 |  | 156 [28.9%] |
| Rash | 92 [90.2%] | 70 [84.3%] | 0.3279 |  | 486 [90%] |
| Mouth ulcers | 85 [83.3%] | 76 [91.6%] | 0.1502 |  | 491 [90.9%] |
| Coryza | 33 [32.4%] | 33 [39.8%] | 0.3722 |  | 165 [30.6%] |
| Cough | 35 [34.3%] | 32 [38.6%] | 0.6571 |  | 146 [27%] |
| Breathlessness | 7 [6.9%] | 1 [1.2%] | 0.0759 |  | 2 [0.4%] |
| Cold peripheries / poor perfusion | 9 [8.8%] | 3 [3.6%] | 0.2306 |  | 2 [0.4%] |
| Vomiting | 49 [48%] | 29 [34.9%] | 0.0996 |  | 59 [10.9%] |
| Poor feeding | 79 [77.5%] | 70 [84.3%] | 0.3217 |  | 303 [56.1%] |
| Diarrhea | 7 [6.9%] | 6 [7.2%] | >0.9999 |  | 17 [3.1%] |
| Constipation | 8 [7.8%] | 2 [2.4%] | 0.1890 |  | 20 [3.7%] |
| Reduced urine output | 24 [23.5%] | 20 [24.1%] | >0.9999 |  | 48 [8.9%] |
| Irritability | 36 [35.3%] | 29 [34.9%] | >0.9999 |  | 36 [6.7%] |
| Lethargy | 65 [63.7%] | 36 [43.4%] | 0.0089 |  | 51 [9.4%] |
| Seizures | 7 [6.9%] | 8 [9.6%] | 0.6755 |  | 0 |
| Reduced limb movement | 6 [5.9%] | 1 [1.2%] | 0.1318 |  | 0 |
| Headache | 14 [13.7%] | 8 [9.6%] | 0.5315 |  | 7 [1.3%] |
|  |  |  |  |  |  |
| **Examination** |  |  |  |  |  |
| Toxic looking | 69 (67.6) | 53 (63.9) | 0.6994 |  | 7 (1.3) |
| Dehydration | 18 [17.6%] | 10 [12%] | 0.3944 |  | 22 [4.1%] |
| Mean peak body temperature (ºC, range) | 38.6  [36.8-40.9] | 38.1  [36.8-40.5] | <0.0001 |  | 37.2  [36.4-39.8] |
| Mean peak body temperature ≥ 38.5ºC | 60 [58.8%] | 32 [38.6%] | 0.0094 |  | 20 [3.7%] |
| Mean heart rate  (beats per min, range) | 132 [72-219] | 124 [80-160] | 0.0038 |  | 123 [80-238] |
| Mean heart rate >150/min | 12 [11.8%] | 1 [1.2%] | 0.0069 |  | 6 [1.1%] |
| Rash | 97 [95.1%] | 78 [94%] | 0.9915 |  | 516 [95.6%] |
| Mouth ulcers | 90 [88.2%] | 78 [94%] | 0.2785 |  | 496 [91.9%] |
| Lethargy/drowsy | 35 | 15 | 0.0210 |  | 1 |
| Irritability | 21 [20.6%] | 13 [15.7%] | 0.5025 |  | 0 |
| Limb weakness | 9 [8.8%] | 0 | 0.0046 |  | 0 |
| Neck stiffness | 7 [6.9%] | 1 [1.2%] | 0.0759 |  | 0 |
| History of or witnessed myoclonus | 21 [20.6%] | 9 [10.8%] | 0.1121 |  | 4 [0.7%] |
| Cerebellar signs | 2 [2%] | 0 | 0.5026 |  | 0 |
| Abnormal lung examination | 7 [6.9%] | 3 [3.6%] | 0.5157 |  | 0 |
| Abnormal cardiovascular examination | 4 [3.9%] | 2 [2.4%] | 0.6925 |  | 9 [1.7%] |
| Hepatomegaly | 16 [15.7%] | 10 [12%] | 0.6195 |  | 34 [6.3%] |
| Splenomegaly | 1 [1.8%] | 0 | >0.9999 |  | 4 [0.7%] |
| Vesicle present | 49 [48%] | 39 [47%] | 0.9953 |  | 367 [68%] |
|  |  |  |  |  |  |
| **Viral isolation** |  |  |  |  |  |
| HEV71 isolated | 52 [51%] | 34 [41%] | 0.2258 |  | 173 [32%] |
| CVA16 isolated | 2 [2%] | 9 [10.8%] | 0.0102 |  | 74 [13.7%] |
| Both HEV71 and CVA16 isolated | 0 | 0 |  |  | 14 [2.6%] |
| Other virus isolated | 8 [7.8%] | 11 [13.3%] | 0.3360 |  | 31 [5.7%] |
| Negative isolation | 35 [34.3%] | 24 [28.9%] | 0.5320 |  | 161 [29.8%] |
| No virus isolation done | 5 [4.9%] | 5 [6%] | 0.7551 |  | 87 [16.1%] |
|  |  |  |  |  |  |
| **Laboratory results** |  |  |  |  |  |
| Mean haemoglobin  (g/dL, range) | 11.5 [8.9-15.1] | 11.3 [8.5-13.4] | 0.4866 |  | 11.6  [7.3-17.3] |
| Mean white cell count (cells/μL, range) | 13247  [5200-264000] | 13300  [5700-283000] | 0.9543 |  | 12010  [5100-31100] |
| Mean platelet count (platelelt/μL, range) | 387537  [152000-994000] | 353220  [180000-504000] | 0.1473 |  | 356336 [125000-775000] |
| Mean serum sodium (mmol/L, range) | 138 [130-146] | 137 [131-145] | 0.6392 |  | 138  [131-148] |
| Mean blood glucose (mmol/L, range) | 5.9 [2.8-24.1] | 5.4 [1.9-13.4] | 0.1618 |  |  |
| Median CSF cell count (cells/μL, range) | 41[0-1090] | 2 [0-5] | <0.0001 |  |  |
| CSF neutrophilia | 17 | 0 |  |  |  |
| Median CSF protein concentration (g/dL, range) | 0.28 [0.06-1.26] | 0.18 [0.06-0.57] | <0.0001 |  |  |
| CSF protein concentration > 0.45 | 22 | 2 |  |  |  |
| Mean CSF: plasma glucose ratio | 0.66 [0.2-1.1] | 0.67 [0.14-1.1] | 0.7374 |  |  |
|  |  |  |  |  |  |
|  |  |  |  |  |  |
| Note: |  |  |  |  |  |
| * Comparison between children that had HFMD-CNS and those with HFMD-Non-CNS | | | | | |
| HFMD: Hand, foot, and mouth disease | | | | | |
| CSF: Cerebrospinal fluid | | | | | |
| HFMD-CNS: HFMD with central nervous system complication | | | | | |
| HFMD-Non-CNS: Severe HFMD without central nervous system involvement | | | | | |
| HEV71: Human enterovirus 71 | | | | | |
| CVA16: Coxsackie virus A16 | | | | | |
|  |  |  |  |  |  |
