# Supplementary material for: Identification and validation of clinical predictors for the risk of neurological involvement in children with hand, foot, and mouth disease in Sarawak
Source: BMC Infect Dis. 2009 Jan 19;9:3. doi: 10.1186/1471-2334-9-3 (PMC2637878; doi:10.1186/1471-2334-9-3)
Supplement: Additional file 2 — Clinical features of the 730 children with Hand, foot and mouth Disease that were admitted during the 2006 outbreak according to clinical severity. The clinical features of the children with HFMD-CNS (i.e. with CSF pleocytosis) are compared to those with HFMD-Non-CNS (i.e. no CSF pleocytosis). The clinical features of children with mild HFMD is also included here. [file 1471-2334-9-3-S2.doc]

| Additional File 2. Clinical features of 730 children with hand, foot, and mouth disease that were admitted during the 2006 outbreak according to clinical severity | | | | | |
| --- | --- | --- | --- | --- | --- |
| Severity | HFMD-CNS (with CSF pleocytosis) | HFMD-Non-CNS  (no CSF pleocytosis) | p value* |  | Mild HFMD |
| Number of children | 250 | 186 |  |  | 294 |
| **History** |  |  |  |  |  |
| Median age in months (months, range) | 23.9  (0.3-126.0) | 27.8  (2.2-134.2) | 0.1456 |  | 31.2  (1.6-128.0) |
| Male, no (%) | 152 (60.8) | 97 (52.2) | 0.0878 |  | 163 (54.9) |
| Chinese | 97 (38.8) | 52 (28.0) | 0.0239 |  | 64 (21.5) |
| Iban | 106 (42.4) | 88 (47.3) | 0.3559 |  | 166 (55.9) |
| Malay/Melanau | 40 (16) | 42 (22.6) | 0.1063 |  | 63 (21.2) |
| Fever at home | 247 (98.8) | 178 (95.7) | 0.0610 |  | 253 (85.2) |
| Mean duration of fever  at home (days) | 2 (0-6) | 1.9 (0-7) | 0.2095 |  | 1.3 (0-10) |
| Duration of fever at home ≥ 3 days | 76 (30.4) | 46 (24.7) | 0.2316 |  | 29 (9.8) |
| Mean total duration of fever (days) | 3.6 (0.5-9.5) | 2.7 (0-7.5) | <0.0001 |  | 1.5 (0-10) |
| Total duration of fever ≥ 3 days | 183 (73.2) | 84 (45.2) | <0.0001 |  | 33 (11.1) |
| Past history of HFMD | 2 (0.8) | 0 | 0.5096 |  | 4 (1.3) |
| Had history of contact with children with HFMD | 103 (41.2) | 50 (26.9) | 0.0027 |  | 96 (32.3) |
| Rash | 191 (76.4) | 114 (61.3) | 0.0010 |  | 213 (71.7) |
| Mouth ulcers | 218 (87.2) | 158 (84.9) | 0.5926 |  | 272 (91.6) |
| Coryza | 42 (16.8) | 28 (15.1) | 0.7194 |  | 41 (13.8) |
| Cough | 48 (19.2) | 37 (19.9) | 0.9535 |  | 36 (12.1) |
| Breathlessness | 5 (2.0) | 3 (1.6) | >0.9999 |  | 1 (0.3) |
| Cold peripheries / poor perfusion | 5 (2.0) | 2 (1.1) | 0.7039 |  | 2 (0.7) |
| Vomiting | 62 (24.8) | 35 (18.8) | 0.1710 |  | 14 (4.7) |
| Poor feeding | 203 (81.2) | 154 (82.8) | 0.7625 |  | 185 (62.3) |
| Diarrhea | 11 (4.4) | 10 (5.4) | 0.8066 |  | 8 (2.7) |
| Constipation | 3 (1.2) | 0 | 0.2645 |  | 0 |
| Reduced urine output | 92 (36.8) | 68 (36.6) | 0.9610 |  | 72 (24.2) |
| Irritability | 54 (21.6) | 41 (22.0) | 0.9948 |  | 15 (5.1) |
| Lethargy | 90 (36.0) | 36 (19.4) | 0.0002 |  | 19 (6.4) |
| Seizures | 13 (5.3) | 9 (4.8) | 0.9595 |  | 0 |
| Reduced limb movement | 1 (0.4) | 0 | >0.9999 |  | 0 |
| Headache | 17 (6.8) | 8 (4.3) | 0.3672 |  | 16 (5.4) |
|  |  |  |  |  |  |
| **Examination** |  |  |  |  |  |
| Toxic looking | 52 (20.8) | 14 (7.5) | 0.0002 |  | 1 (0.3) |
| Dehydration | 85 (34.0) | 50 (26.9) | 0.1375 |  | 38 (12.8) |
| Mean peak body temperature (ºC, range) | 38.7  (36.8-40.3) | 38.3  (36.8-40.4) | <0.0001 |  | 37.6 (36.4-39.9) |
| Mean peak body temperature ≥ 38.5ºC | 170 (68) | 83 (44.6) | <0.0001 |  | 42 (14.1) |
| Mean heart rate  (bears per min, range) | 145  (86-204) | 141  (82-209) | 0.0607 |  | 130  (80-199) |
| Mean heart rate >150/min | 105 (42) | 69 (37.1) | 0.3497 |  | 49 (16.5) |
| Rash | 222 (88.8) | 147 (79.0) | 0.0077 |  | 250 (84.2) |
| Mouth ulcers | 235 (94.0) | 181 (97.3) | 0.1605 |  | 287 (96.6) |
| Lethargy | 74 (29.6) | 31 (16.7) | 0.0026 |  | 5 (1.7) |
| Irritability | 37 (14.8) | 34 (18.3) | 0.3997 |  | 6 (2.0) |
| Limb weakness | 2 (0.8) | 0 | 0.5096 |  | 0 |
| Neck stiffness | 17 (6.8) | 4 (2.2) | 0.0438 |  | 0 |
| History of or witnessed myoclonus | 176 (70.4) | 91 (48.9) | <0.0001 |  | 31 (10.4) |
| Abnormal lung findings | 19 | 6 | 0.0828 |  | 1 |
| Abnormal cardiovascular findings | 1 (0.4) | 1 (0.5) | >0.9999 |  | 0 |
| Hepatomegaly | 20 (8) | 7 (3.8) | 0.1064 |  | 4 (1.3) |
| Splenomegaly | 3 (1.3) | 1 (0.5) | 0.6394 |  | 0 |
| Vesicle present | 41 (16.4) | 22 (11.8) | 0.2281 |  | 61 (20.5) |
|  |  |  |  |  |  |
| **Viral isolation** |  |  |  |  |  |
| HEV71 isolated | 53 | 46 |  |  | 58 |
| CVA16 isolated | 0 | 0 |  |  | 0 |
| Other HEV isolated | 12 | 19 |  |  | 13 |
| Negative isolation | 123 | 114 |  |  | 148 |
|  |  |  |  |  |  |
| **Pan EV PCR** |  |  |  |  |  |
| positive | 163 (65.2) | 133 (71.5) |  |  | 188 |
| negative | 57 (22.8) | 18 (9.7) |  |  | 35 |
|  |  |  |  |  |  |
| **HEV71 specific PCR** |  |  |  |  |  |
| positive | 96 (38.4) | 60 (32.3) |  |  | 83 |
| negative | 63 (25.2) | 88 |  |  | 87 |
|  |  |  |  |  |  |
| Number of HEV71  positive children | 114 | 73 |  |  | 104 |
| Number of HEV71 negative children | 109 | 109 |  |  | 144 |
| Note: |  |  |  |  |  |
| * Comparison between children that had HFMD-CNS and those with HFMD-Non-CNS | | | | | |
| HFMD: Hand, foot, and mouth disease | | | | | |
| CSF: Cerebrospinal fluid | | | | | |
| HFMD-CNS: HFMD with central nervous system complication | | | | | |
| HFMD-Non-CNS: Severe HFMD without central nervous system involvement | | | | | |
| HEV71: Human enterovirus 71 | | | | | |
| CVA16: Coxsackie virus A16 | | | | | |
|  |  |  |  |  |  |
